# Practical Measures for Dealing With the Struggles of Nurses Caring for People With Amyotrophic Lateral Sclerosis Comorbid With Cognitive Impairment in Japan

**DOI:** 10.3389/fpsyg.2021.752461

**Published:** 2021-09-16

**Authors:** Mitsuko Ushikubo, Emiko Nashiki, Tadahiro Ohtani, Hiromi Kawabata

**Affiliations:** ^1^Graduate School of Health Sciences, Gunma University, Maebashi, Japan; ^2^Nursing Department, Gunma University Hospital, Maebashi, Japan; ^3^Nursing Department, Mihara Memorial Hospital, Isesaki, Japan

**Keywords:** amyotrophic lateral sclerosis, cognitive impairment, comorbid, nursing, neuroscience, care

## Abstract

Amyotrophic lateral sclerosis (ALS) is a devastating neurodegenerative disease for which there is currently no cure. This study aimed to explore the situations with which nurses struggled, their implemented practical measures, and the challenges they experienced when caring for patients with ALS comorbid with cognitive impairment (hereinafter, targeted patients). In this qualitative study, we conducted a survey with nurses (*n* = 121) experienced in caring for ALS patients; the survey contained a free-writing section in which participants described their struggles regarding care delivery for these patients. To collect data on practical measures that nurses had already implemented or wanted to propose regarding care delivery for the targeted patients, we conducted four focus group interviews (*n* = 22). We used a qualitative inductive approach to extract the categories. Fifty-eight nurses (49.6%) completed the free-writing survey section. The situations in which nurses struggled in care for the targeted patients were organized into three categories: “Patients’ strong persistency on specific requirements for nursing assistance in their daily lives,” “Patients’ problematic behaviors toward nurses,” and “Struggles in communicating with and understanding patients’ wishes.” Nurses reported these situations as stressful, and they affected care quality. The practical measures implemented when caring for the targeted patients were organized into five categories: “Cognitive impairment assessment,” “Care delivery to deal with patients’ strong persistency on specific requirements for assistance in their daily lives,” “Communication,” “Supporting the decision-making process,” and “Collaboration between the hospital and the community.” Multidisciplinary collaboration in the hospital, and collaboration between the hospital and the community from an early stage is necessary to share the results of the assessment and diagnosis of cognitive impairment. Our evidence underlines that guideline and care manual establishment may lead to improved care delivery and to the unification of care deliveries to respond to patients’ strong persistency.

## Introduction

The prevalence of neurodegenerative diseases increases with age (De Ronchi et al., 2005). Recent data shows that the number of patients with amyotrophic lateral sclerosis (ALS) has been increasing, albeit it remains a rare disease (Talbott et al., 2016). ALS is a devastating neurodegenerative disease that affects the motor system, being characterized by progressive neuro-deterioration (Miller et al., 2009). It can evoke upper and lower limb dysfunction, dysphagia, and speech impairments, making activities of daily life arduous (Hobson and McDermott, 2016). Unless tracheostomy positive pressure ventilation (TPPV, i.e., invasive mechanical ventilation, which requires delivering breath through a tracheostomy tube) is initiated for dealing with patients’ respiratory failure, ALS patients usually die within a 3- to 5-year period (Miller et al., 2009).

Meanwhile, the prevalence of multimorbidity among older adults is expected to increase in the near future. From 2015–2035, the proportion of individuals with more than four diseases is expected to almost double, and two-thirds of the patients are expected to have some sort of cognitive impairment and depression (Kingston et al., 2018).

Despite the traditional view that ALS is a neurodegenerative disease that does not impact cognition, it is often comorbid with cognitive impairments (Goldstein and Abrahams, 2013). Specifically, research shows that approximately 50% of ALS patients have cognitive dysfunction and 10–15% have dementia (Lomen-Hoerth et al., 2003). Frontotemporal dementia (FTD) has received much attention from scholars studying dementia in ALS patients, with up to 15% of FTD patients and 30% of ALS patients experiencing overlapping features (Lomen-Hoerth, 2011).

Numerous scholars have studied on either care delivery for people with cognitive impairment or for people with ALS. Caring for a person with either dementia or ALS is mentally and physically demanding (Bromberg et al., 2011; Maayan et al., 2014), and caring for a person with both is considered to be even more challenging. Psychological stress in people who provide care for ALS may impact well-being in ALS patients and, possibly, management of symptoms in ALS patients (Siciliano et al., 2017).

However, few studies have focused on care for the people who care for those who have ALS and cognitive impairment, even though there are numerous pathological, clinical, and genetic studies. The burden of care for caregivers of a person with both ALS and FTD is high (Lillo et al., 2012; Cui et al., 2015; Caga et al., 2019). Nurses, as professionals in both medicine and care, support many informal caregivers and collaborate with certified home caregivers on a long-term basis in delivering care for the ALS patients with cognitive impairment.

Moreover, Japan was shown to have a higher rate of patients with ALS undergoing TPPV when compared with other developed countries, and a subset of these Japanese patients was shown to extend their lives for more than 10 years (Tagami et al., 2014). The motor impairment severity may be associated with cognitive impairment severity (Chiò et al., 2019).

With advance in medical technology and care as well as the advent of a super-aged society, the number of ALS patients with comorbid cognitive impairment is expected to increase. To ensure that these patients receive optimal care, we see the need for clearly understanding the current status of nursing care delivery for ALS patients with comorbid cognitive impairment. The purpose of this study was to clarify the situations with which nurses struggled, effects of situations nurses struggled with in caring for these patients on the quality of care, the practical measures implemented or proposed by nurses, and the challenges they experienced when caring for patients with ALS comorbid with cognitive impairment.

## Materials and Methods

### Design

This study was a cross-sectional, qualitative descriptive study in which a questionnaire survey and focus group interviews were conducted. In order to collect a wide range of data from a large number of nurses, we thought that a questionnaire survey method with a free-writing section would be suitable. Following this survey, focus group interviews (FGI) were conducted to collect a rich dataset, because focus groups facilitate open discussion sharing of nurses’ experiences and opinions.

### Sample and Data Collection

We conducted a cross-sectional survey using an anonymous questionnaire in November 2018. Through purposive sampling, we recruited 172 nurses experienced in care delivery for ALS patients from two hospitals, 38 home visiting nursing stations, and 11 public health centers in one prefecture in the Kanto region. Regarding questionnaire distribution, head nurses distributed them to hospital nurses, with potential participants having been asked to complete the questionnaire and return it in a collection box; meanwhile, home health and public health nurses received and returned the questionnaire by mail. The questionnaire asked participants to indicate their characteristics (workplace, years of nursing experiences, and the number of patients with ALS they have supported) and fill in the freewriting section on situations in which nurses often struggle to deliver care for ALS patients with comorbid cognitive impairment.

In total, we collected 117 valid questionnaires, with 58 of them containing responses in the free-writing section (response rate: 49.5%). Among participants, 32 (55%) worked in a hospital, 19 at home visiting nursing stations (33%), and 7 (12%) in public health centers that care for patients with intractable neurological diseases. The mean number of years of nursing experience was 16 years (range: 6 months to 32 years). Ten nurses (17%) cared for one to five ALS patients, 14 nurses (24%) cared for six to ten patients, and 34 nurses (57%) cared for more than 11 ALS patients.

One year after a questionnaire survey, FGI were conducted to collect data on the care delivery experience and practical knowledge of nurses experienced in caring for the targeted patients. FGI is a qualitative methodology used to conduct in-depth data collection through group dynamics; it enables scholars to obtain detailed data inaccessible through quantitative research methods, as well as broad and dynamic data inaccessible through solo interviews (Anme, 2001). The participants were recruited from the same facilities as in the questionnaire survey. The directors or hospital head nurses informed their staff about the study. The inclusion criterion of FGI was to have experience in support for patients with ALS. In total, 23 individuals expressed interest in participating in the focus group interviews. However, one nurse could not attend to the interview because of an emergency call. Accordingly, 12 home care nurses, five hospital nurses, one nursing consultant, two nursing educators, one neurologist, and one medical social worker were included in the study. One participant was in her 30s, and all the others were in their 40s or 50s. All but one of the participants were female. To avoid uneven distribution of work locations, they were divided into four groups, with each group ranging from five to six participants.

Prior to discussion onset, the first author shared the results of the questionnaire survey (i.e., on situations with which nurses struggles in caring for the targeted patients) with the participants in order to get the discussion started smoothly and to have participants review the survey results. Each group was moderated by T.O., H.K and two other research team members who were a nurse certified by the Japanese Society of Intractable Disease Nursing.

The moderator followed an interview guide, which included the following questions: “Introduce yourself and, if you have already faced any situations with which you struggled regarding care delivery for ALS patients comorbid with cognitive impairment aside from those in the survey results report, share them with us;” “Share practical measures you have implemented, may want to propose, and discuss what would be better strategies;” and “Share any challenges you perceive regarding care delivery for ALS patients when they have a comorbid cognitive impairment.” The moderators raised follow-up questions based on participants’ responses and discussions; each focus group interview lasted approximately 60 min. The data source was the record made by the scribe, supplemented by each moderator of the groups to make it easier to read or understand.

### Data Analysis

We conducted descriptive statistics for examining the following participants’ characteristics: the number of nursing care experiences, the number of ALS patients they delivered care for, and their workplaces. We used qualitative inductive analysis for assessing the responses to the free-writing survey section. This qualitative data analysis was carried out based on the method proposed by Greg (2007), which aims to clarify actual situation from the emic viewpoint, and is suitable for this study.

The procedure of the analysis was as follows. All responses were reviewed several times to obtain the sense of the whole. The first author broke down each description into one sentence with one meaning. These descriptions were categorized by assessing their similarities and differences to extract subcategories. Similar subcategories were grouped together to derive categories. All co-authors examined the first author’s analysis processes separately, and repeatedly discussed and confirmed the appropriateness of the descriptions, subcategories, and categories. Afterward, all authors scrutinized the relationship between situations and their influences on care quality, and expressed these relationships visually through a diagram.

Similarly, we used the qualitative inductive approach for the FGI records. The records from each group were merged for each question. Each description was shortened to one sentence with one meaning, ensuring that no meaning would be lost. Those descriptions were then categorized into subcategories and categories based on their similarities and differences.

### Rigor

Trustworthiness or rigor of a study refers to the degree of confidence in data, interpretation, and methods used to ensure the quality of a study (Polit and Beck, 2014). This study employed constant comparative analysis to ensure trustworthiness for analysis of both the survey data and the interview data. The codes, subcategories, and categories were repeatedly scrutinized back and forth. The contents and classification of the categories were discussed with the authors with a master’s degree or Ph.D. degree who had substantial experience with qualitative-inductive analysis and further validated by an experienced researcher. In addition, member checking was employed for the survey analysis. Member checking, participant validation is a technique for exploring the credibility of results (Birt et al., 2016). The report followed the Standards for Reporting Qualitative Research (O’Brien et al., 2014).

### Ethical Considerations

This study was approved by the ethics committee of the institution to which the first author is affiliated. On the cover of the questionnaire in the survey, participants were informed study aims, methods, and ethical considerations. Specifically, they were assured that confidentiality would be maintained, participation and withdrawal from research were entirely voluntary, and that informed consent would be considered to have been granted with the return of the questionnaire. For the FGI, before their onset, we explained the same items to potential participants and obtained informed consent from all the participants.

## Results

### Situations With Which Nurses Struggled in Care for People With ALS Comorbid With Cognitive Impairment

The data analysis for the free-writing section in the survey yielded three categories, nine subcategories, and 52 descriptions. Hereinafter, subcategories are denoted by ⟨⟨⟩⟩, and descriptions are shown in ⟨⟩ (Table 1). We describe each category below, with explanation by using some main descriptions.

**TABLE 1:** Situations with which nurses struggle in caring for people with ALS comorbid with cognitive impairment.

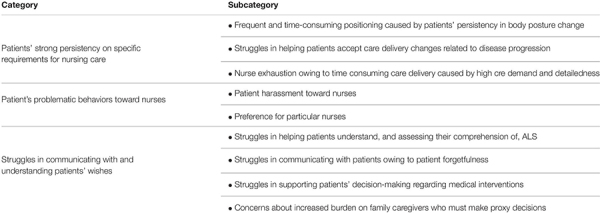

#### Category: Patients’ Strong Persistency on Specific Requirements for Nursing Care

This category consisted of three subcategories. The first was ⟨⟨Frequent and time-consuming positioning caused by patients’ persistency in body posture change⟩⟩, which was extracted from four descriptions; ⟨It takes a lot of time to get some patients to be satisfied with the body positioning⟩, ⟨Some patients are unusually picky about changes in their body position⟩, ⟨The frequent demands for repositioning from one ALS patient interferes with nurses’ care delivery for other patients⟩, and ⟨Some patients frequently press the nurse call button to request fine, millimetric repositioning no matter what time⟩.

The second subcategory was ⟨⟨Struggles in helping patients accept care delivery changes related to disease progression⟩⟩, which was extracted from five descriptions including, ⟨They have a strong desire to eat and drink, despite the risk of aspiration⟩, and ⟨Some patients want to use the regular toilet even if they are fully dependent on assistance⟩.

The third subcategory was ⟨⟨Nurse exhaustion owing to time-consuming care delivery caused by high care demand and detailedness⟩⟩, which was extracted from seven descriptions; for example, ⟨The burden of care is so high that nurses become exhausted⟩, ⟨Nurses take much time to satisfy patients’ requests because these patients are very detailed in their care requirements⟩, and ⟨Nurses spend so much time responding to the request of one patient that they have no time to care for others⟩.

#### Category: Patients’ Problematic Behaviors Toward Nurses

This category consisted of two subcategories. The first was ⟨⟨Patient harassment toward nurses⟩⟩, which was extracted from four descriptions; ⟨There is rejection and verbal abuse toward unexperienced nurses⟩, ⟨Some patients treat nurses like idiots⟩, ⟨Some patients harass nursing staffs they do not like⟩, and ⟨Several nursing staff are experiencing psychogenic symptoms owing to harassment from patients⟩.

The second subcategory was ⟨⟨Preference for particular nurses⟩⟩, which was extracted from three descriptions; for example, ⟨Some patients interfere with the nursing care of other patients owing to preferring a particular nurse⟩, and ⟨Some patients require to change the nurse, because they don’t like that nurse⟩.

#### Category: Struggles in Communicating With and Understanding Patients’ Wishes

This category comprised four subcategories. The first subcategory was ⟨⟨Struggles in helping patients understand, and assessing their comprehension of, ALS⟩⟩, which was extracted from three descriptions; ⟨I had to explain to the patient the nature of one’s disease many times because of one’s poor understanding⟩, ⟨They stubbornly believe that they will be able to do what they were able to do before they got sick.⟩, and ⟨They cannot understand that ALS causes dysphagia and gait problems.⟩.

The second subcategory was ⟨⟨Struggles in communicating with patients owing to patient forgetfulness⟩⟩, which was extracted from 13 descriptions, including ⟨They struggle to convey what they want owing to their forgetfulness⟩, and ⟨They have difficulty selecting characters on the communication board owing to patient forgetfulness, even though their hands can point out letters⟩.

The third subcategory was ⟨⟨Struggles in supporting patients’ decision-making regarding medical interventions⟩⟩, which was extracted from nine descriptions; for example, ⟨We need to consider dangerous actions, such as deliberate self-extubation, to support the decision-making process⟩.

The fourth subcategory was ⟨⟨Concerns about increased burden on family caregivers who must make proxy decisions⟩⟩, which was extracted from four descriptions, such as ⟨We thought that the cognitive decline and the progression of ALS would make it difficult for family caregivers to confirm the patient’s preferences for ventilator use, which would lead to an increased burden on the family⟩.

**FIGURE 1:**
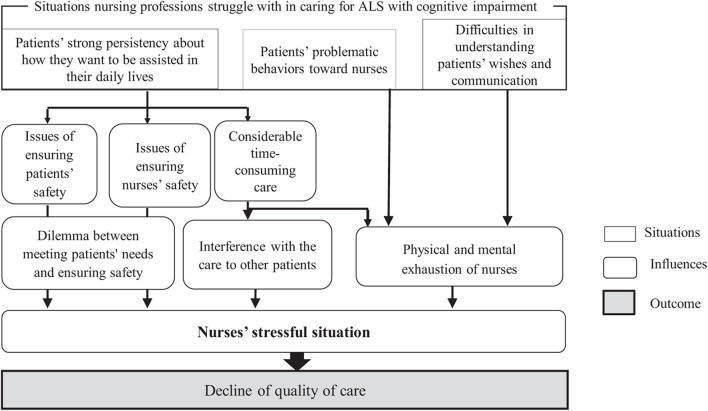
Effects of situations nurses struggle with in caring for the patients with ALS and cognitive impairment on the quality of care.

We further clarified how these three situations affected care quality by carefully examining each description, extracting the content that indicates the influence on care. These situations evoked dilemmas for nurses, as they were caught between meeting patients’ needs and ensuring patient safety. They also interfered with care delivery to other patients, evoked the physical and mental exhaustion of nurses, and led to an increasing number of stressful situations for nurses. Hence, these situations may have had a negative effect on care quality, as shown in Figure 1.

### Practical Measures Implemented or Proposed by Nurses

Regarding practical measures implemented or proposed by nurses for care delivery to patients with ALS comorbid with cognitive impairment, we extracted five categories, 13 subcategories, and 33 descriptions (Table 2).

**TABLE 2:** Participants’ reports on practical measures to care for ALS patients comorbid with cognitive impairment.

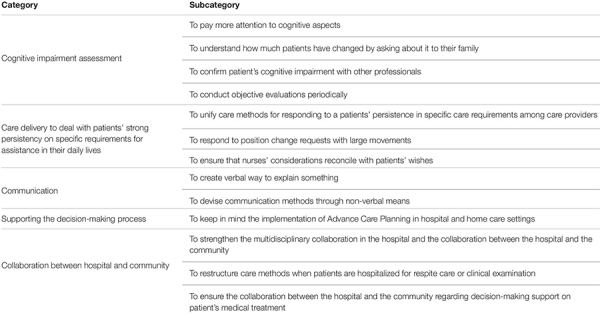

#### Category: Cognitive Impairment Assessment

This category comprised four subcategories. The nurses mentioned that they paid attention to changes in both physical and cognitive aspects and collected information on patients’ cognitive impairment from a multifaceted perspective; specifically, from the perspective of family members and other professionals. Additionally, they mentioned conducting objective cognitive assessments periodically.

#### Category: Care Delivery to Deal With Patients’ Strong Persistency on Specific Requirements for Assistance in Their Daily Lives

This category comprised three subcategories. The first was ⟨⟨To unify the care methods for responding to a patient’s persistence in specific care requirements among care providers⟩⟩, which was extracted from two descriptions; ⟨The contents of care to be provided should be unified among nursing staffs⟩, and ⟨It would be good to create a manual to unify the care method among all care providers⟩. The second was ⟨⟨To respond to position change requests with large movements⟩⟩, which was extracted from one description, ⟨It is a good practice to move the patients significantly, before making fine, millimetric adjustments⟩. The third subcategory was ⟨⟨To ensure that nurses’ considerations reconcile with patients’ wishes⟩⟩, which was extracted from one description, ⟨We need to discuss with the patients, in advance, about when and the amount of time we can be available for them, and acquire their consent⟩.

#### Category: Communication

This category comprised two subcategories: ⟨⟨To create a verbal way to explain the cognitive impairment⟩⟩, and ⟨⟨To devise communication methods through non-verbal means⟩⟩. If nurses tell the patients that they suffer from dementia, the patients may express that they no longer want to go to the hospital for examination, so home care nurses try to talk to them creatively. An example would be something like, ⟨People of your age need to check for brain atrophy owing to aging⟩. Nurses also mentioned that, ⟨Verbal explanations alone will not solve the problem, so we use gestures and facial expressions, creating non-verbal ways to explain something⟩.

#### Category: Supporting the Decision-Making Process

This category comprised one subcategory: ⟨⟨To keep in mind the implementation of advance care planning in hospital and home care settings⟩⟩. As the nurses experienced ⟨⟨Struggles in supporting patients’ decision-making regarding medical interventions⟩⟩, they described the following: ⟨We need to give opportunities for family members to discuss about patients’ wishes before cognitive impairment onset⟩ and ⟨We need to support the decision-making processes and connect with the community⟩.

#### Category: Collaboration Between the Hospital and the Community

This category comprised three subcategories: ⟨⟨To strengthen the multidisciplinary collaboration in the hospital and the collaboration between the hospital and the community⟩⟩, ⟨⟨To restructure care methods when patients are hospitalized for respite care or clinical examination⟩⟩, and ⟨⟨To ensure the collaboration between the hospital and the community regarding decision-making support on patient’s medical treatment⟩⟩. The participants remarked that in hospitals, unlike home care settings, they deal with numerous patients, hindering the ability of hospital staff to respond to patients’ individual wishes in detail. Still, the hospital setting was also described to be a facilitator of nurse-physician collaboration. In this situation, patients often have little choice and are forced to accept nurses’ requests to change care methods.

Moreover, the nurses described that, while most patients stay in hospitals for short periods, hospital staff can be involved intensively in and available for 24 h a day to caring for patients. Accordingly, our study participants remarked that the strength of hospital care should be utilized to assess patients’ cognitive function level. They also emphasized the need to restructure care methods upon patient hospitalization for clinical examination or respite care; they believed that this may help to ensure that the care delivered is relevant to one’s physical independence level. For this to be operationalized, they remarked the need for a greater collaboration between the hospital and the community.

### Challenges in Care for ALS Patients With Comorbid Cognitive Impairment

Two categories were extracted from the analysis of FGI records: challenge of integrating *cognitive impairment assessment* into ALS care and challenge of *unification of nursing staff to respond patients in the same way*. The former was extracted from one subcategory, ⟨⟨Challenges to implement the cognitive impairment assessment⟩⟩. This subcategory had two descriptions: ⟨The content of neuropsychological tests is too demanding and I am not comfortable conducting them⟩, and ⟨When family members are asked to perform neuropsychological tests at home, it is necessary to consider the influence of the family’s psychological situation⟩.

The second category comprised two subcategories. One subcategory was ⟨⟨Challenges in responding to and experiencing dilemmas regarding patient requests among nurses⟩⟩, which was extracted from four descriptions, including ⟨There are many times when the nursing staff disagree on how to care for the patient⟩, ⟨There are nurses who have a strong sense of mission to care for ALS patients and try to meet all their needs to any extent⟩, and ⟨The nurses always feel uncomfortable and face dilemmas about meeting all patient requests⟩. The second subcategory was ⟨⟨Challenges in responding to requests from other care providers to comply with the patient’s requests⟩⟩, which was extracted from two descriptions; ⟨Nurses may struggle to deal with the family members who wish to fulfill patient’s requests that seem unreasonable⟩, and ⟨Nurses may often be requested to care for the patient’s needs and reminded that this is the responsibility of nurses by other care providers, although it may be difficult to deal with patients’ needs⟩. The value of people involved are diverse, and unifying them is a heavily difficult task.

## Discussion

This study clarified that the situations in which nurses struggled to care for the targeted patients were related to the following: patients’ strong persistency on specific requirements for nursing assistance in their daily lives, patients’ problematic behaviors toward nurses, and struggles in communicating with and understanding patients’ wishes. These situations increased nurses’ stress and may have facilitated the deterioration of care quality. Moreover, we extracted five practical measures to deal with these some problems and two challenges that nurses may experience. Those findings were integrated into the following discussion for better care of ALS patients with cognitive impairment.

### Need to Increase Awareness of Nurses Regarding Cognitive Impairment in ALS

In this study, the participants reported struggling with patients’ persistency on specific requirements regarding their nursing care. Indeed, the non-adherence to treatment of individuals with comorbid ALS and cognitive impairment was shown to be twice as high as that of ALS without dementia (Cui et al., 2015). Thus, caregivers of such patients often experience a heavy care burden (Lillo et al., 2012; Caga et al., 2019). Moreover, FTD was shown to be associated with personality changes, irritability, poor insight, perseverance, obsession, disinhibition, and altered social conduct (Phukan et al., 2007). Despite the scientific evidence on these associations, on the practical setting, nurses may interpret some of these behaviors as stubborn expressions of patients’ autonomy (Bromberg and Bromberg, 2017), potentially not recognizing that the patients experience not only ALS but also a cognitive impairment. Therefore, we suggest for nurses to pay more attention to the possible presence of FTD in ALS patients.

In concordance with this suggestion from our study, a past research described that, in clinical settings, Japanese neurologists should pay more attention to the cognitive and behavioral aspects of patients with ALS (Watanabe et al., 2020). Another study conducted with Japanese nurses clarified that, although 60% of them were aware of the cognitive impairments associated with ALS, they only learned about this within the last 1 or 2 years and through their own clinical experience (Ushikubo et al., 2021). Therefore, it seems that both nurses and neurologists need to pay more attention to cognitive function in ALS patients.

### Conducting Appropriate Assessment of Cognitive Impairment in Delivering Care for ALS Patients

Our study participants struggled with patients’ problematic behaviors, such as harassment. In relation to this finding, a prior study has assessed coping strategies of neurology nurses experiencing abuse from patients and families, identifying that over 96% of the nurses reported some form of verbal abuse and over 60% reported some form of physical abuse; to deal with this abuse, nurses utilized avoidance coping strategies (Trahan and Bishop, 2016). The literature recommends that nursing professionals should develop nursing practice that eliminates the use of avoidance as a way of dealing with abuse or these problematic behaviors (Trahan and Bishop, 2016). Thus, we see a critical need for assessing patients with ALS for cognitive impairment; this may support nurses, other healthcare professionals, and informal caregivers by ensuring that they have more information on and are better prepared to care for these patients. This may help reduce the stress surrounding patient care in such complex settings.

Hence, it seems important for nurses to acquire skills that allow for them to evaluate different types and levels of cognitive impairment. On the topic, the literature recommends professionals to use several batteries of evaluations for ALS-FTD, which include the Mini-Mental State Examination (Folstein et al., 1975), the Frontal Assessment Battery (Dubois et al., 2000), the Japanese version of the ALS-FTD Questionnaire (Watanabe et al., 2016), and the Montreal Cognitive Assessment (MoCA) (Nasreddine et al., 2005). Of these, the MoCA has been regarded as the most sensitive clinical scale for evaluating cognitive impairment in ALS (Nagashima et al., 2019). Meanwhile, in our study, the nurses pointed out cognitive impairment assessment as a challenge, reporting many barriers to this end. For example, they observed that the motor (e.g., hand weakness, dysarthria) or verbal communication deficits often found in ALS patients may interfere with the performance of cognitive impairment tests. Furthermore, the application of a screening test may upset or even offend some patients, and so careful consideration is required.

### Understanding Patients’ Wishes in the Communication Difficulties

Moreover, nurses struggled to communicate with and to understand patients’ wishes. When dealing with ALS patients, healthcare professionals often need to introduce treatments in a timely manner to ensure symptom alleviation and optimize conditions (Hogden et al., 2021). To ensure that this is done according to patient’s wishes, ALS patients need to understand their medical conditions and be able to decide about their medical treatment. Health care professionals identified that the patient’s acceptance of the disease and its progression by both patient and caregiver were one of the most important factors to support decision-making (Martin et al., 2016). Surrounding this topic, a prior study showed how the inability of patients to communicate may lead to their family members making proxy decisions, placing a great burden on the family (Foley and Hynes, 2018). To deal with this issue, the nurses in our current study reported attempting to communicate with patients through verbal and non-verbal methods. Thus, we suggest the need to provide specialized communication support for nurses dealing with both cognitive impairment and bulbar symptoms of ALS patients. Multidisciplinary collaboration, including occupational and speech therapists may be essential in such settings (De-Bernardi-Ojuel et al., 2021). Furthermore, past research remarked the need to integrate advanced care planning ever since the diagnosis, as this may allow for family caregivers to be relieved from the burden of making proxy decisions and for patients to get involved in the decision-making process regarding their medical treatment (Bromberg and Bromberg, 2017).

### Seamless Care With Multifaceted Perspective and Multidisciplinary Cooperation Between Hospital and Community

This study showed that community collaboration is a potential practical measure to be used by nurses for both decision-making process about medical interventions and care method changes. Based on our participants’ remarks, collaboration between the hospital and the community and multidisciplinary collaboration is essential to understand the patient’s value, preferences, and goals to support their advance care planning (Seeber et al., 2019).

In the home care setting, nurses are easier for listen to the true feelings of the patients. However, it is also easier for patients to escalate their demands and home care nurses experience difficulties in asking patients for changes in care delivery. Therefore, the optimal way for delivering care to such patients may be to use a seamless, multifaceted perspective and multidisciplinary cooperation. This may allow for stakeholders to use the strengths of both the hospital and the community and to cover for their respective weaknesses.

### Limitation

There were several limitations in this study. The FGI was very active, so we could have used a longer timeframe, which could ensure that we reached data saturation. In addition, to determine whether there are other situations with which nurses struggle with and whether nurses utilize other types of practical measures, we suggest for future studies to include larger sample sizes and a sample that is representative of various Japanese regions. As ALS is a rare disease as designated by the Japanese government and it is even more difficult to secure a sufficient number of nurses with experience in supporting ALS patients with cognitive impairment, this study employed purposive sampling to efficiently secure participants. Furthermore, the sampling was limited to the region as this research planned to conduct FGI following the questionnaire survey. We recommend to conduct a national survey or to recruit participants with the cooperation of several regional nursing associations or home care nurse councils. Those may be able to secure a larger number of participants. Even if participants are dispersed all over the country, it is feasible to hold FGI or individual interviews as online meetings grow in popularity.

### Conclusion

This qualitative study clarified three types of situations with which nurses struggled during the care for people with ALS comorbid with cognitive impairment, as well as five categories of practical measures. Further, nurses deemed that the cognitive impairment assessment for ALS patients and the unification of care deliveries to respond patients’ strong persistency were challenging issues. The multidisciplinary collaboration between the hospital and the community may be important to ensure that the diagnosis of cognitive impairment is appropriately shared between stakeholders and to ensure that they are aware of the condition of these patients. In addition, all nurses need to respond to patients’ excessive demands and patients’ harassment to nurses in same way. The people involved with patients have different care policies, such as establishing a standard care for all, under their various views, but their unification is important. This study suggests to establish care guideline or care manuals, which may lead to the provision of an optimal care. It needs to include cognitive impairment assessment, unification of care delivery to respond to patients’ strong persistency, coping for problematic behavior to nurses, communication that covers both language and cognitive impairments caused by ALS, and the decision-making support, by collaborating between hospital and community.

## Data Availability Statement

The original contributions presented in the study are included in the article/supplementary material, further inquiries can be directed to the corresponding author/s.

## Ethics Statement

The studies involving human participants were reviewed and approved by the Ethical Review Board for Medical Research Involving Human Subjects in Gunma University. The patients/participants provided their written informed consent to participate in this study.

## Author Contributions

MU conceptualized the research, designed the study, gathered and analyzed the data, and drafted the manuscript. EN, TO, and HK contributed with data collection, data analysis, and a critical revision of the manuscript. All authors contributed to the article and approved the submitted version.

## Conflict of Interest

The authors declare that the research was conducted in the absence of any commercial or financial relationships that could be construed as a potential conflict of interest.

## Publisher’s Note

All claims expressed in this article are solely those of the authors and do not necessarily represent those of their affiliated organizations, or those of the publisher, the editors and the reviewers. Any product that may be evaluated in this article, or claim that may be made by its manufacturer, is not guaranteed or endorsed by the publisher.
